# Resolving repeat families with long reads

**DOI:** 10.1186/s12859-019-2807-4

**Published:** 2019-05-09

**Authors:** Philipp Bongartz

**Affiliations:** Heidelberg Institute for Theoretical Studies, Schloss-Wolfsbrunnenweg 35, Heidelberg, 69118 Germany

**Keywords:** Genome assembly, Repeat families, Repeat resolution

## Abstract

**Background:**

Draft quality genomes for a multitude of organisms have become common due to the advancement of genome assemblers using long-read technologies with high error rates. Although current assemblies are substantially more contiguous than assemblies based on short reads, complete chromosomal assemblies are still challenging. Interspersed repeat families with multiple copy versions dominate the contig and scaffold ends of current long-read assemblies for complex genomes. These repeat families generally remain unresolved, as existing algorithmic solutions either do not scale to large copy numbers or can not handle the current high read error rates.

**Results:**

We propose novel repeat resolution methods for large interspersed repeat families and assess their accuracy on simulated data sets with various distinct repeat structures and on drosophila melanogaster transposons. Additionally, we compare our methods to an existing long read repeat resolution tool and show the improved accuracy of our method.

**Conclusions:**

Our results demonstrate the applicability of our methods for the improvement of the contiguity of genome assemblies.

**Electronic supplementary material:**

The online version of this article (10.1186/s12859-019-2807-4) contains supplementary material, which is available to authorized users.

## Background

Long read sequencing technologies [1–4] have brought us almost within reach of perfect genome assemblies. For circular bacterial genomes, full resolution is already considered as being the current standard for assemblers that are based on long-read sequencing technologies [5]. Perfect bacterial genome assemblies are achieved by spanning repeat elements with reads that are long enough to be anchored in unique sequences on both sides of the repeat [6]. However, eukaryotic organisms generally contain interspersed repeat families, mostly transposons, that are responsible for repetitive regions that are not spanned by the current read lengths. In complex genomes, these interspersed repeat families are the most prevalent reason for assembly breaks [7–9]. Especially in plant genomes contiguity of assemblies is often limited by a high number of interspersed repeats [10, 11]. Frequently, most interspersed repeats originate from but a few repeat families [12]. As the number of indistinguishable repeat copies grows, it becomes increasingly unlikely to find a unique path through an assembly graph. Thus, the only strategy to resolve a given repeat family directly from the sequencing data is to detect distinguishing features between the copies of a repeat family. Several approaches to detect and utilize such repeat differences have been proposed [13–15]. However, these existing repeat resolution methods are geared toward 2-10 repeat copies. This limits their applicability to only a small subset of repeat structures as they occur in complex genomes [7, 8].

Here, we present a method that is similar to that of Tammi [14], in that it also uses multiple sequence alignments (MSA) and a statistical analysis of the MSA columns to determine discriminative variations. It uses more sophisticated clustering heuristics to overcome the limitation of Tammi’s method to an error rate below 11%, and to repeat families with 10 or less copies. For simulated data sets with distinct repeat structures we are able to resolve repeat families with 100 copies under the typical PacBio error rate of 15%, while assuming an absolute number of repeat copy differences comparable to that of other methods. Our analysis of Drosophila melanogaster transposons proves that similar results can be achieved with empirical data, while our comparison to an existing repeat resolving tool for long read data demonstrates the improved accuracy (82.9% vs 50.6% resolved copies) and reduced runtime of our method.

## Methods

### Data sets

#### Simulating repeat data

To avoid overfitting our method to one specific repeat family structure, we use three different approaches to create simulated repeat families with ≥x% difference between copy pairs.

Equidistant Simulations: In *equidistant* simulated repeats, each copy has x/2% variants that distinguish it from the initial template. In pairwise comparisons these per-copy differences then yield a difference of x%.

Distributed Variants Simulation: Additionally, we conduct a *distributed* variant repeat family simulation. Here, we distribute each variant over a subset of copies. Thus, each copy consists of an intersection of variants. In turn, these variants characterize a subset of copies, rather than a single copy. Adding 3x% variants again yields an expected difference between copy pairs of x% (see Additional file 1: S4).

Tree-like Simulations: Finally, we simulate *tree*-like variant repeats. Here we create a repeat family by building a binary tree of copies, each copy obtaining x/2% variants that distinguish it from the parent copy. The leaves of this tree create a repeat family where sister leaves show a difference of x%. The binary tree simulates a simplified version of the phylogenesis of repeat families via copying and mutation [16, 17].

Our three simulation scenarios pose distinct algorithmic challenges in variant detection and copy disambiguation. In general, a repeat resolving method should perform well under all three simulation scenarios. To benchmark our algorithms, we create synthetic data sets for each scenario described above. Each simulated data set contains 100 copies derived from a randomly created 30 kbp template. This is the repeat length, where spanning reads become unlikely with current read lengths. In practice, repeat regions of this size or larger will often, but not always, consist of several distinct repeat modules. This does not impact the applicability of our methods.

These 100 copies are diversified with equal numbers of substitutions, insertions and deletions of single bases. We create data sets with 0.1%, 0.5% and 1% minimal copy differences respectively. To each copy we add two unique 10kbp flanking sequences on both sides of the 30kbp repeat. From these copies 30-40X coverage is randomly sampled, with the read length distribution and coverage modelled after the empirical PacBio data set described in the following paragraph [18]. The 10 kbp flanking sequences ensure that the coverage does not decrease at the ends of the repeat sequence. Each read exhibits the typical PacBio error rate of 11.5% insertions, 3.4% deletions and 1.4% substitutions. (For more details on the simulated data sets, see Additional file 1: Table S7).

#### Transposon data sets

As simulated data is often less challenging to analyse than real data, we also test our algorithms on several empirical PacBio data sets obtained from a subline of the ISO1(y;cn,bw,sp) strain of Drosophila melanogaster [18]. Each data set is created by selecting reads that fully map to a transposon template. These templates are taken from the canonical transposon sequence set [19], with a length cutoff of > 4 kbp, as resolving even shorter repeat sequences is not required due to current read lengths. There are seventeen transposon data sets numbered from 0 to 21, with the missing numbers indicating transposons below the length cutoff. The transposon template length varies between 4.4 kbp and 7.5 kbp with a mean of 5.8 kbp and a median of 5.3 kbp, the copy numbers lie between 7 and 157. Due to the selection of reads that fully fit the template, the initial sequencing coverage of 90x is reduced to 35-54x. (For more details on each transposon data set, see Additional file 1: Tables S6 A) and B)). The ground truth for the resolution of each repeat family is manually determined by clustering the flanking sequences of every transposon data set according to the Levenshtein distance [20].

### Resolving repeat families

Our repeat resolution algorithm consists of several steps. First, we calculate a multiple sequence alignment to accurately compare the reads sampled from copies of the repeat family. We proceed to extract variations between repeat copies by a statistical analysis of intra-alignment column deviations. On the basis of these extracted variations, we conduct a two-step clustering. We subdivide the reads covering a section of the repeat by determining strong signals within the variations and apply a simple clustering algorithm on the subdivided sets of reads. Then, we apply an algorithm that utilizes the resulting clusters to resolve long repetitive stretches in the genome, that can only be covered by several adjacent reads. In the following we describe each step in detail.

#### Multiple sequence alignments

In a pre-processing step, the simulated reads are arranged into a multiple sequence alignment (MSA) [21]. This initial MSA is computed by aligning all reads to a repeat family template. In our test data sets we use simulated repeat templates and templates extracted from existing genome assemblies, but in practice any sequence of a repeat family of interest can be used. Ideally the MSA is initiated by a consensus sequence of the entire repeat, however, a long raw read can also be used for this purpose. The initial MSA is subsequently refined by realigning all sequences until the sum of pairwise alignment scores does not further improve. This approach is similar to the method proposed by Anson [22].

#### Detecting significant bases

Due to the high error rate, each site of this refined MSA contains all four bases as well as coverage and alignment gaps. To find the sites where this variation can be explained by significant differences between repeat copies that are beyond random error, we conduct a statistical analysis of the co-appearance of bases at different MSA sites. Every base *b* at site *j* defines a group $G^{b}_{j}$ containing the sequences that have a *b* at site *j*. The likelihood of statistically independent $G^{b2}_{j} \cap G^{b1}_{i}$ exceeding a certain size is described by the cumulative hypergeometric probability [13, 14]. The lower the probability of a given intersection of base groups, the more likely it is that both groups are defined by a significant variation between repeat copies. Via pairwise comparison of all base groups we can subsequently extract all groups *G* that in at least one comparison exceed a probability cut-off. This probability cut-off is calculated as the inverse of the number of comparisons. In extremely large MSAs the pairwise comparison can be restricted to sites, that contain a majority of bases, as opposed to alignment gaps. This reduces runtime by almost two orders of magnitude. But in the data sets used for this paper, the quadratic complexity of this step does not yet constitute a computational bottleneck.

#### Refining base groups

If a base group *G* extracted from the MSA was completely error free, we could model it as a union of true copy groups *T*_*i*_ with *i*∈*I*_*G*_. Here *T*_*i*_ contains exactly those reads sampled from repeat copy number *i* and *I*_*G*_ describes which copies comprise the base that defines the base group *G*. Due to the existing error rate, *G* will contain a fraction *p* of these true positives in the *T*_*i*_s with *i*∈*I*_*G*_ and also, a fraction *q* of the sequences in the *T*_*i*_s with *i*∉*I*_*G*_ as false positives.

In the following, we describe a framework to refine such groups and to identify those, where the refinement induces a low proportion *q* of false positives and a high proportion *p* of true positives. In this analysis, we assume that all groups have been restricted to contain only sequences that show no coverage gaps on any of the MSA sites from which the groups are derived.

First, we calculate a clique *C* of *n* groups *G*_*j*_, with *j*∈*J* and |*J*|=*n*, that share the most significant positive intersection with *G*. A positive intersection is an intersection that is larger than expected by chance. The parameter *n* is chosen empirically. This is a clique in the graph that contains groups as nodes and statistically significant intersections between those groups as edges. Now we can define a consensus group *C*_*k*_:={*s*|*s*∈*G*_*j*_ for *j*∈*J* with |*J*|>*k*} for every cut-off *k*≤*n*. The cut-off *k* determines in how many groups of the clique a given read has to occur, to be included in the consensus group *C*_*k*_. If the groups that constitute a clique all share the same *I*_*G*_, that is, they all describe the same ground truth group, the following formula gives the probability that a specific read is in the consensus group *C*_*k*_:

$$\sum\limits^{n}_{l< k} \sum\limits_{i+j=l} \text{Pr}(i,l,p)\times\text{Pr}(j,n-l,q)$$

This formula is a sum over the probabilities that a given read occurs in exactly *l* out of *n* groups, with *l*>*k*. A given read occurs in exactly *l* groups, if it occurs in *i* groups as true positive, that is, an element of the *T*_*i*_ with *i*∈*I*_*G*_, and in *j* groups as false positive while *i*+*j*=*l*. These probabilities are given by Pr(.,.,.), the probability mass function of the binomial distribution, which takes as parameters the probabilities *p*,*q* of a group element being a true positive or a false positive, respectively.

**Fig. 1:**
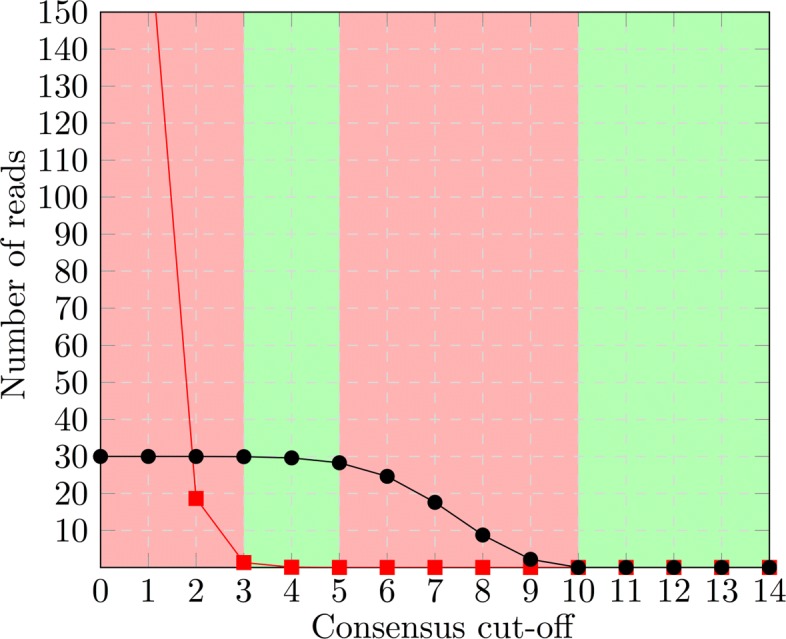
True positives and false positives for a single *T*_*i*_. If the groups *G*_*j*_ of a clique all describe a single *T*_*i*_, the number of false positives (red squares) coming from other groups *T*_*j*_ decreases quickly, while the number of true positives (black dots) remains constant until the cut-off value is relatively high. In the green areas, the cut-off guarantees to yield a consensus that either perfectly contains *T*_*i*_ or is completely empty

The fraction of false positives in *C*_*k*_ coming from the *T*_*i*_ with *i*∉*I*_*G*_, is described by the cumulative probability function ${\sum \nolimits }^{n}_{i=k+1} {n\choose i} q^{i} (1-q)^{n-i} $ of the binomial distribution. As shown in Fig. 1, this fraction of false positives decreases quickly with increasing cut-off *k*, while the number of true positives remains constant for larger *k*. This is due to *p* being significantly larger than *q*. In reality, the subset of *T*_*i*_s described by the groups that form a clique can vary considerably. Also, not every *T*_*i*_ is described by either all or none of the groups. If we consider the *T*_*i*_s separately, we find that if a *T*_*i*_ is contained in *m* groups of the clique, we expect the fraction ${\sum \nolimits }^{m}_{l>k} {\sum \nolimits }_{i+j=l} \text {Pr}(i,l,p)\times \text {Pr}(j,n-l,q)$ of the elements of *T*_*i*_ to occur in the consensus group *C*_*k*_. In this formula *l* is the number of groups, in which an element occurs. This number is split into *i* true positives in the *m* groups that describe *T*_*i*_, and *j* false positives in the groups not describing *T*_*i*_. For low cut-offs *k*, this fraction is close to 100% and we expect all elements of *T*_*i*_ to occur in *C*_*k*_. As *k* increases the expected number of true positives decreases to zero. So, for every *T*_*i*_ there are three separate value ranges for the cut-off *k*, the *perfect* range, in which all elements are contained, the *dropping* range, in which the number of true positives decreases, and the *zero* range, where no elements of *T*_*i*_ are part of *C*_*k*_, any more. See Fig. 1 for an illustration.

**Fig. 2:**
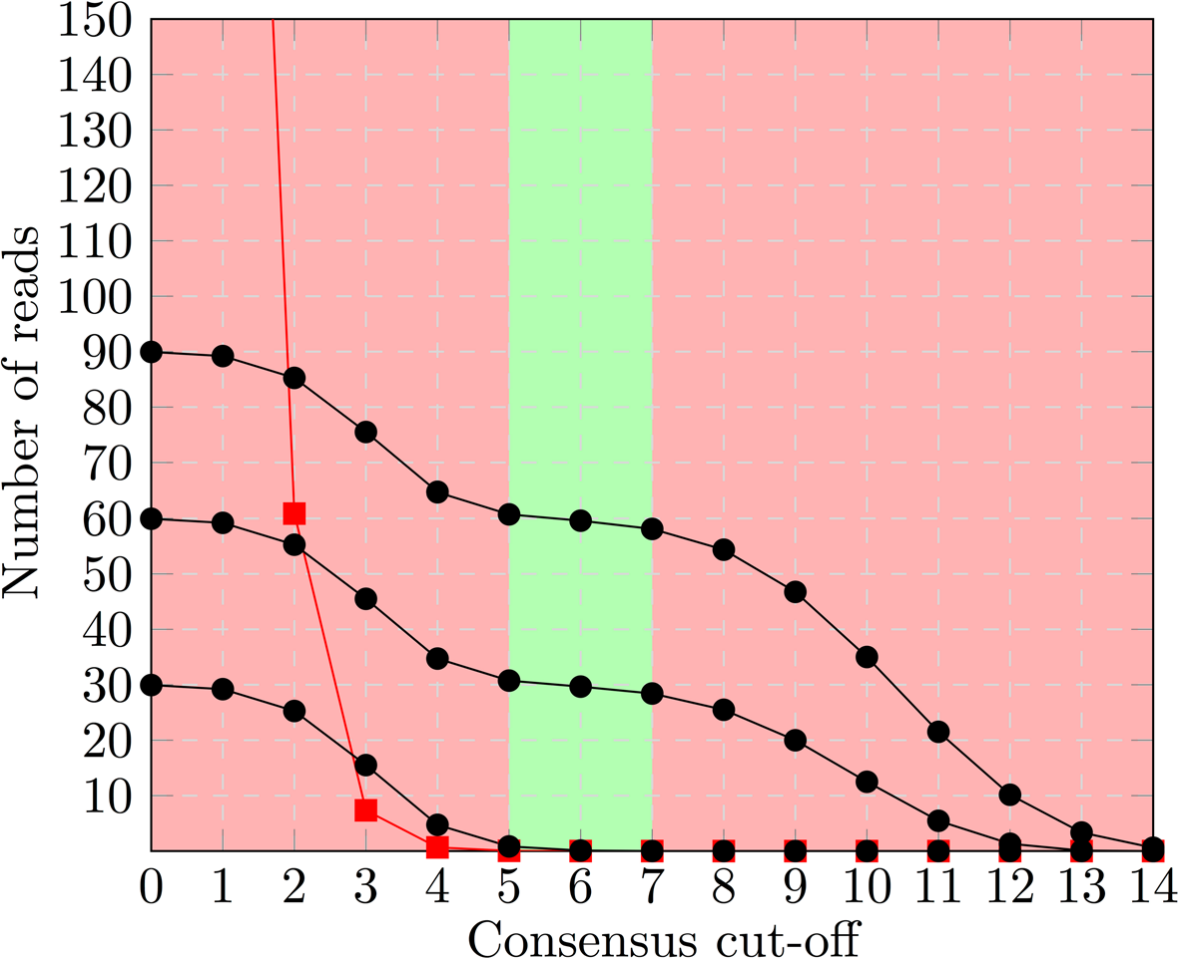
True positives and false positives for several *T*_*i*_s. If the groups *G*_*j*_ of a clique describe several *T*_*i*_, the size of the consensus is determined by the aggregate of the true positives (black dots) from each *T*_*i*_, as well as the false positives (red squares) from the remaining *T*_*j*_s. The green area shows the cut-offs that create a consensus that accurately distinguishes one subset of s from the rest. It is exactly this range where the *perfect* or *zero* ranges of all *T*_*i*_ overlap. Furthermore, the aggregate number of true positives (denoted by the uppermost black dots) stays constant in this range

For distinct *T*_*i*_s the *k* value ranges for perfect and dropping accuracy will be different, due to different values of *m*, the number of groups describing *T*_*i*_. If *k* is high enough for the number of false positives from the *T*_*i*_s *not* described by any clique members to decrease to zero, the number of elements of *C*_*k*_ is equal to the sum over the cardinalities of *T*_*i*_∩*C*_*k*_ as given above. As we will see, minimizing the difference between *C*_*k*_ and *C*_*k*+1_ allows us to determine the optimal cut-off value *k*, which places most *T*_*i*_s into, or close to, either their *perfect* or *zero* range (See Fig. 2).

We call the size difference between *C*_*k*_ and *C*_*k*+1_ the *drop-off* between *C*_*k*_ and *C*_*k*+1_. The size of the *drop-off* is determined by the number of *T*_*i*_s for which the cut-off value *k* is in the *dropping* range. Therefore, a drop-off close to zero indicates that all *T*_*i*_s are either completely contained in *C*_*k*_ or not contained therein at all. The drop-off allows to determine the optimal cut-off value *k* for every clique of groups. More importantly, it allows to rank the different clique consensuses by their likelihood of perfectly describing a subset of *T*_*i*_s.

#### Clustering

The refinement procedure described above aims to extract sufficiently strong signals to accurately classify the sequences into two subsets of copy versions. It can then be applied recursively to each of the respective subsets. For a recursive subdivision to work, it needs to be highly accurate. Otherwise, noise will accumulate in subsets, yielding subsequent analyses increasingly difficult. We achieve this increases accuracy in the recursion via the refinement and the drop-off precision estimate for each refinement. The recursive subdivision terminates, once no subset is left that can produce a consensus group which is sufficiently refined for further subdivision.

When the recursive subdivision process has terminated, we apply a simple clustering algorithm to each of the remaining subsets. It assigns reads to centroids according to the differences that are significant for the subset. To that end, we initially recalculate the statistical significance of each variation restricted to that subset. Only those variations, that still show statistically significant intersections of their base groups, are then used for clustering. For each read we extract the instances of these still significant variations into a so-called read signature. Then, every signature is corrected with the four most similar other signatures for noise reduction and subsequently used as a centroid. In the first round of clustering, signatures are assigned to centroids by the best fit according to the Hamming distance. This creates a large number of clusters of varying size. Some clusters will have fewer elements than half the expected sequencing coverage. We resolve these small clusters by merging their elements into other clusters, again according to the smallest Hamming distance between the signature and the centroid.

#### Resolution

The output of the recursive division step and the subsequent clustering consists of groups of reads that are required to resolve a repetitive region. To resolve a large repetitive region in a genome, we likely have to subdivide our MSA into several sections, whose reads are clustered separately. This keeps the number of reads that completely cover each section high. Together, these sections and their clusterings cover the entire repeat. Initially, however, we examine a simplified one-clustering scenario with some of the reads of each repeat copy having a unique flanking sequence on the 5^′^ end and the other half having unique flanking sequences on the 3^′^ end. We now answer the question how many of these flanking sequences we can accurately connect using the clustering information.

We propose a model to calculate a confidence score for each possible connection. This can be used as a basis for a resolution that takes the probability of mis-assemblies into account as opposed to just providing a "best guess". In the one-clustering scenario it can also be used to assess how well a clustering corresponds to the ground truth. The calculated clusters can be seen as hubs which are entered by incoming reads and can be exited by outgoing reads. We can, for instance, sample a random path from one flanking sequence cluster to another flanking sequence cluster on the other side of the repetitive region. This is done by randomly choosing shared reads that connect the current hub to the next (see Additional file 1: Figure S1). We use the probability of such a randomly sampled path to connect two flanking sequence clusters to define a unidirectional connection confidence. The full connection confidence is then calculated as the product of the unidirectional connection confidences in both directions. It is normalized such that the connection confidences of all possible connections for a flanking sequence cluster sum to 1.0. Naively, calculating the fraction of randomly sampled paths that start from a 5^′^ flanking sequence and end in a 3^′^ flanking sequence, has a time complexity that is exponential in the number of clusterings. To address this, we break down the calculation into clustering-to-clustering path probability matrices that can be multiplied to give the probability of a complete path. This is possible, because the probability of reaching a specific cluster from a given cluster, is independent of the path taken to the given cluster.

Let *X*_*i*<*n*_ be the 3^′^-flanking sequence clusters and *Y*_*i*<*m*_ be the 5^′^-flanking sequence clusters, while $H^{k}_{i< n_{k}}$ denotes the 0<*k*≤*l* calculated clusterings of sections of the repetitive sequence that lie in between. The following matrices describe the probability of a randomly chosen read from one particular cluster to connect to a read from a cluster that is part of the next clustering.

$$I_{ij} = \frac{\vert X_{i} \cap H^{1}_{j} \vert}{\vert X_{i} \vert} \in \mathbb{Q} \cap \left[ 0,1 \right] $$

$$O_{ij} = \frac{\vert Y_{i} \cap H^{l}_{j} \vert}{\vert H^{l}_{j} \vert} \in \mathbb{Q} \cap \left[ 0,1 \right] $$

$$C^{k}_{ij} = \frac{\vert H^{k}_{i} \cap H^{k+1}_{j} \vert}{\vert H^{k}_{i} \vert} \in \mathbb{Q} \cap \left[ 0,1 \right] $$

The probability of a longer path can then be calculated by multiplying the connection probabilities between all clusters along the path. The overall connection probability is calculated by summing over the connection probabilities of all possible paths. In the one clustering scenario, the path possibilities are given by the different clusters that can be taken, so the probability of connecting *X*_*i*_ and *Y*_*k*_ is $P_{ik}= {\sum \nolimits }_{j< n_{1}}I_{ij} \times O_{jk}$. In matrix notation *P*=*IO*. For longer paths we can inductively expand this probability matrix for any number of clusterings. Thus, the overall probability of connecting *X*_*i*_ to *Y*_*k*_ for *l* clusters *H*^*k*^ is *P*=*IC*^1^*C*^2^…*C*^*l*^*O*. This stepwise path-probability calculation reduces runtime complexity to *l* matrix multiplications. The matrix size does not exceed the estimated number of repeat copies. In the multi-step calculation of connection confidence, we analogously multiply the path-probability in both directions and normalize, so that the confidences of all possible connections of a flanking sequence cluster sum to 1.0.

## Results

We integrated the algorithms for realigning, detecting significant variants, calculating drop-off consensus groups, subsequent hierarchical subdivision, final clustering, and connecting flanking sequences, into our repeat resolution tool. We test it on nine simulated data sets, one for each combination of the minimal copy differences 0.1%, 0.5%, 1% and the repeat structures equidistant, distributed and tree-like (see 2.1.1). We also conduct experiments on 17 empirical transposon data sets (see 2.1.2.). To assess the resolution of isolated clusterings, we use the one-step resolution algorithm introduced above. Here the ground truth groups are used to replace both flanking clusterings. That means, we calculate the connection confidence from a ground truth cluster to itself, via the calculated clustering. If the calculated clustering does not differentiate between two ground truth groups of sequences, each of these groups will not correctly or unambiguously be connected to itself. We call two ground truth groups *connected*, if for both groups the other group provides the connection with the highest connection confidence. If this connection is correct, that is, if the two connected groups are identical, we say that the ground truth group or copy group is *resolved* by the calculated clustering. If the connected ground truth groups are different groups, we call the connection a *false positive*. For the simulated data sets we additionally calculate the connection confidences from one flanking clustering to the other flanking clustering via the calculated clustering in between using the multi-step resolution algorithm. Here, we call two flanking clusters *connected*, if, for both clusters, the other group provides the connection with the highest connection confidence. A copy group is likewise called *resolved*, if both flanking clusters belonging to the copy group are *connected* to each other. The multi-step resolution is necessary for the practical feasibility of our clustering algorithm. We compare the single-step resolution of the simulated MSA sections with the single-step resolution of the transposon MSAs. We additionally examine the relationship between single-step resolution results and multi-step resolution results to assess the applicability of our algorithms to very long repeats.

### Simulated data sets

**Fig. 3:**
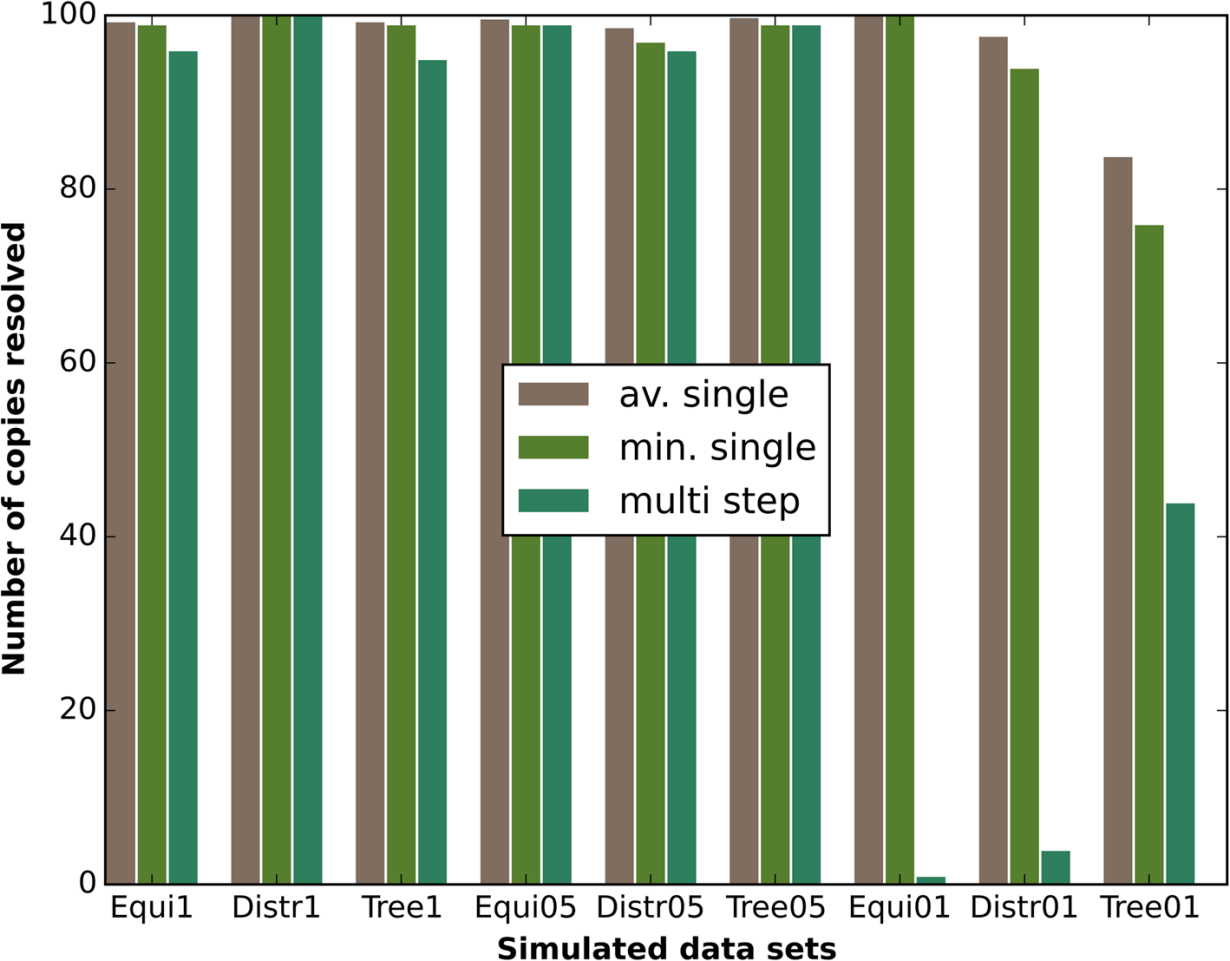
Single-step resolution for simulated data. We compare the average number of resolved copies of the single-step resolutions, the minimal number of resolved copies of the single-step resolution and the number of resolved copies of the multi-step resolution for all simulated data sets

To make the single-step resolution of our simulated data sets comparable to the transposon data sets, we divide each MSA in six non-overlapping sections that approximately contain 5 kbp of repeat sequence. We then compute clusterings for each of these sections separately. We additionally use these six clusterings to calculate a resolution for the entire repeat, that is, we determine which flanking sequence clusters are connected by applying the multi-step algorithm, see (2.2.5). Figure 3 shows that for both single-step resolution and multi-step resolution the number of resolved copies is high (≥ 95%) for all data sets with 0.5% or 1% minimal copy differences. In fact, only the distributed data sets show an appreciable decline in resolved copies between the 1% and 0.5% copy difference data sets. The number of resolved copies for the single-step resolution of the 0.1% copy difference data sets remains high. However, with just 0.1% minimal copy differences the clusterings are not accurate enough to support robust multi-step resolution. Only the tree-like data set with 0.1% copy differences can still resolve more than 40% of its copies over the entire repeat length.

**Fig. 4:**
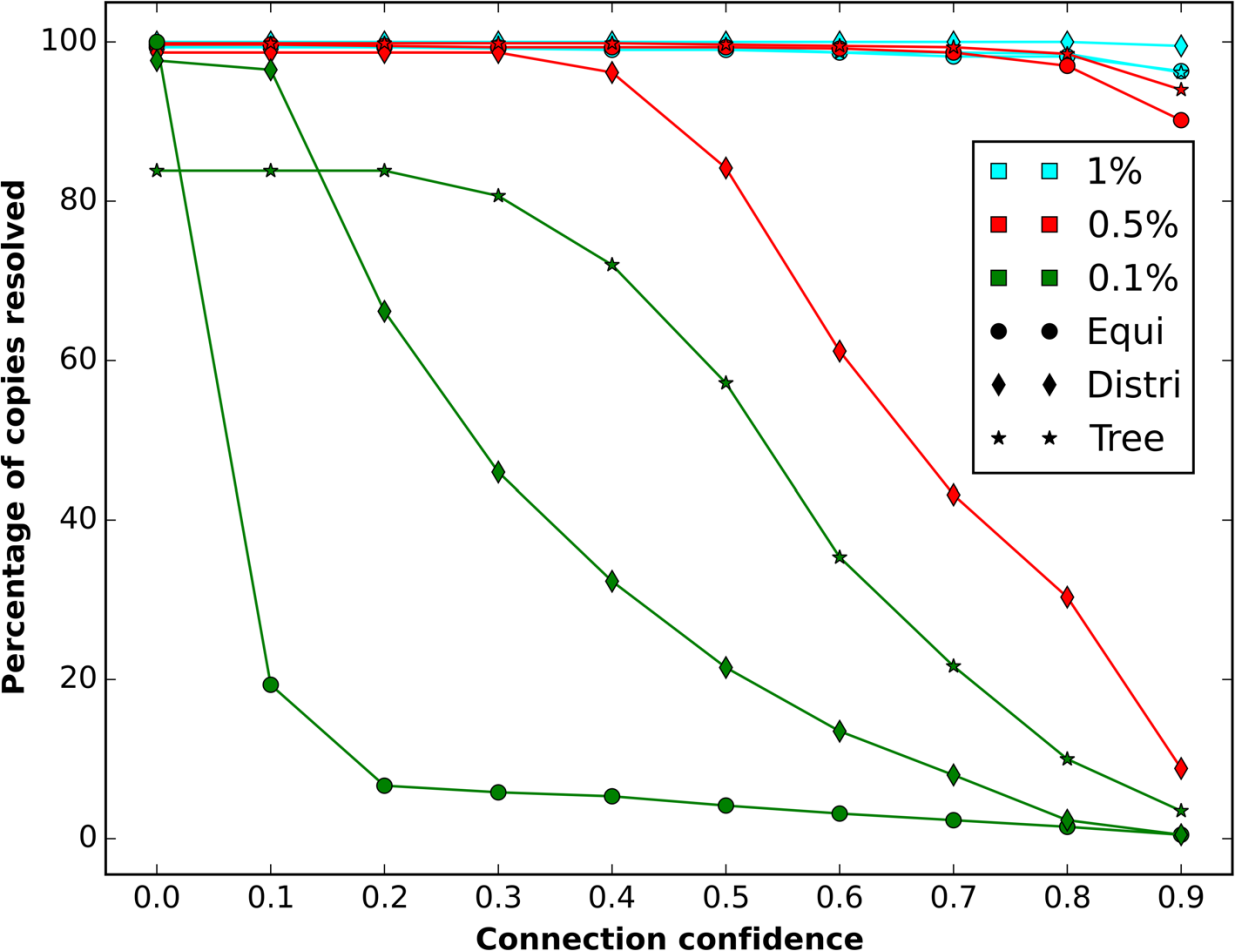
Resolution by connection confidence for simulated data. For all simulated data sets the average number of resolved copies of the single-step resolution above a specific connection confidence

This failure of the multi-step resolution can be predicted from the single-step connection confidences. In Fig. 4 we see, that for data sets with just 0.1% minimal copy differences the connection confidences are generally very low, with the exception of the tree-like data set. The tree-like data set however, has on average only 84% resolved copies in the single step resolution. The fraction of unresolved copies compounds over six resolution steps, which explains the 44% multi-step resolved copies for that particular data set.

The simulated data sets show that multi-step resolution results depend on two properties of the single step resolutions: The fraction of unresolved copies and the connection confidence. In particular, resolved copies with a connection confidence below 0.2 seem not to support the multi-step resolution. It is worth noting that there were no false positives in either single-step resolutions or multi-step resolutions.

### Transposon data sets

**Fig. 5:**
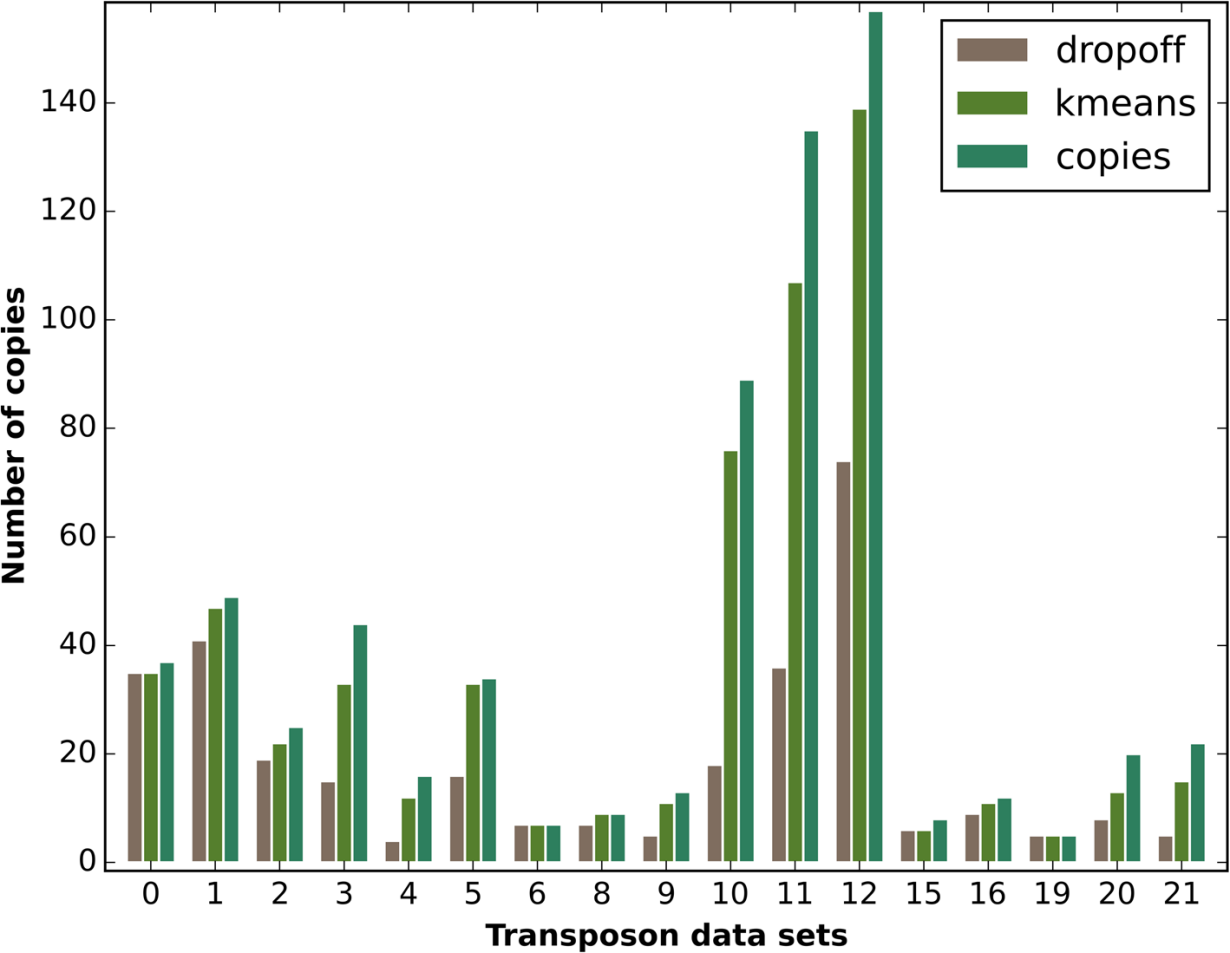
Transposon resolution results. This figure compares the number of resolved copies for the dropoff subdivision algorithm and for the full algorithm with the additional kmeans-like clustering to the total number of copies in each transposon data set

The simulated data sets are created in a way, such that the information necessary to obtain a full resolution is available in each data set. Evidently, we cannot expect to replicate these results for empirical data. Some transposon families will be too highly conserved, while others will contain at least some members that have only recently diverged and did not yet accumulate sufficient differentiating mutations. Moreover, the ground truth copy groups have been obtained by clustering the unique flanking sequences. This process is unlikely to provide a completely accurate ground truth, as is available for the simulated data. Instead copy groups that have not been accurately resolved in the ground truth will add noise to the assessment process, worsening the apparent results. Despite these limitations, according to the metrics introduced above, in all transposon data sets at least 65% of the copy groups are resolved, while only a single data set shows false positives. Several of the smaller data sets are perfectly resolved, while some of the larger data sets are almost perfectly resolved with 35 out 37, 33 out of 34, and 47 out of 49 copy groups being correctly resolved respectively. The three largest groups with 89, 135 and 157 copies respectively, are resolved by more than 84% on average. Moreover, the initial refinement step with recursive subdivision already resolves more than 50% of all copies. This shows that it constitutes an essential part of the clustering algorithm, see Fig. 5.

These are surprisingly good results, that come, however, with a caveat. Many of these copy groups are resolved only due to very few or just a single and noisy distinguishing variant. The results on simulated data show that as few as 5 differences (0.1% of 5 kbp) between repeat copies still allow for single-step resolution. However, they also indicate that the resulting clusterings do not necessarily support the multi-step resolution that is necessary to resolve transposons of a realistic length. To put our results into perspective, we assess the number of statistically significant differences between transposon copies. To this end, we utilize the ground truth information to create consensus signatures for each copy group, that consist of the most common base for that copy group in each statistically significant column in the respective MSA. We then compare these consensus signatures to each other to obtain an estimate of the number of differences between transposon copies.

This analysis shows that the percentage of copies that differ from all other copies by at least *n* bases drops quickly with increasing *n*, (see Additional file 1: Figure S2). This decrease is mirrored by the number of resolved copies that have a connection confidence above a specific threshold, (see Additional file 1: Figure S3). Around 45-55% of the copies exhibit sufficient differences. Consequently, this is the percentage of transposon copies that achieve a high enough connection confidence to support multi-step resolution. This would be a relatively low number, if the copies with high connection confidence were to be different for every resolution step. However, given that the number of differences is a result of the evolutionary history shared by the entire sequence of a copy, it is likely that the resolvable copies will tend to remain unaltered for each section of the MSA. We observe this in the transposon data, where the minimal number of differences to other copies in the first half of a copy and the minimal number of differences in the second half, tend to correlate significantly for most data sets (median Pearson correlation 0.483). This correlation, and the total number of differences, is likely to increase with larger MSA sections.

### Comparison with competing method

In this section we compare our methods against the long read assemblers Canu and MARVEL, and against the dedicated repeat resolving method split_dis that is part of the Daccord package [23].

Full-scale long read assemblers, like Canu and MARVEL, do not use repeat resolution methods that go down to specific differences in the repeat copies. This fundamentally limits their repeat resolution capabilities. In MARVEL, repeat resolution is restricted to the use of spanning reads and the detection of unique combinations of repeat modules [7], making the resolution of a 30 kbp repeat family challenging for MARVEL.

Canu has a dedicated repeat resolution step that is based on alignment score differences of corrected reads. In the original Canu paper [5] this resolution step of Canu and the resolution capability of FALCON are benchmarked in a very similar fashion as we do on our simulated datasets: A PacBio read datasets is simulated with a 30 kbp repeat and subsequently assembled. For Canu, the simulated dataset cannot be assembled contiguously for repeat differences below 3%, for FALCON [24] this threshold is 5%. The parameter differences between this benchmark and our simulated data, with 2 repeat copies versus our 100 repeat copies, 12% read error versus our 15% read error, and 3% copy differences versus the 1% or less we use, decrease the chances of Canu resolving our simulated data.

To adapt our simulated data sets for the requirements of genome assemblers, we modified our simulated datasets with 1% copy differences, to model contiguous sequences (instead of just repeat sequence with flanking sequence), and ran Canu on the reads sampled from these sequences. As expected, all three types of simulated datasets were assembled into roughly one hundred contigs, indicating an unsuccessful repeat resolution, (see Additional file 1: S8).

We also executed MARVEL on all three simulated datasets, similarly resulting in roughly one hundred disjointed contigs, (see Additional file 1: S8 and Figure S9). These results confirm Canu’s earlier repeat resolution assessment and MARVEL’s methodological limitations.

However, there exists one dedicated repeat resolution tool for PacBio data called split_dis. The split_dis tool [15] is part of the Daccord package [23]. The Daccord package contains a suite of tools for processing long reads, centered around the read correction program daccord. The processing necessary for executing split_dis involves computing local alignments with daligner [25], calling a corrected consensus version of each read using the daccord program, and computing quality values for the bases of each read. Split_dis then filters the local alignments for each read separately and retains only those that do not exhibit differences that are likely to be associated with copy differences. By finally selecting only those overlaps of the processed read, where the retained local alignments span almost all of the overlapping region, we generate a list of “true” overlaps for the read. In the following we will refer to this entire repeat resolving pipeline as *Daccord*, while we refer to our pipeline as *RepeatResolver*.

According to its author, using Daccord is not feasible for repeat families with significantly more than 10 copies (German Tischler, personal communication, 12.9.2018). Our results confirm this limitation, as the runtime per read ranges from minutes for the transposon data sets with fewer than 10 copies, to hours for data sets with copy numbers between 10 and 20, and days for transposon data sets with more than 20 copies (see Additional file 1: Table S6 B)). For comparison, the entire RepeatResolver pipeline takes 2 hours for transposon data set 2, which has 25 repeat copies. This amounts to 0.1 minutes per read as opposed to 1 day and 19 hours required by Daccord. As the data sets contain between 257 and 7317 reads (see Additional file 1: Table S6 B)) this makes the application of Daccord infeasible for large repeat families. Accordingly, we assess performance only for transposon data sets with less than 30 copies.

Daccord computes a list of overlaps for each read, while RepeatResolver yields a clustering of reads. To compare the results, we transform the RepeatResolver output into Daccord-style output, by assigning to each read all the reads in the same cluster as overlaps.

In the comparison, we only consider reads for which ground truth information is available. We test three methods of overlap selection to achieve the highest possible resolution accuracy for Daccord. We 1.) select the 20 longest overlaps, 2.) select the 20 longest local alignments provided by Daccord, 3.) choose the 20 local alignments with the best alignment score, among those local alignments that span more than 90% of the overlapping region. We compare the RepeatResolver result to the best result among these three methods for each data set.

Selecting substantially more than 20 overlaps reduces accuracy, as more than 30 reads with ground truth information are not always available for each repeat copy. Selecting less than 20 overlaps does not increase accuracy. The average number of reads per RepeatResolver cluster with ground truth information varies from 14 to 62 between data sets. They cluster close to the number of 20 chosen for the Daccord overlaps, see Additional file 1: Table S6 B).

**Fig. 6:**
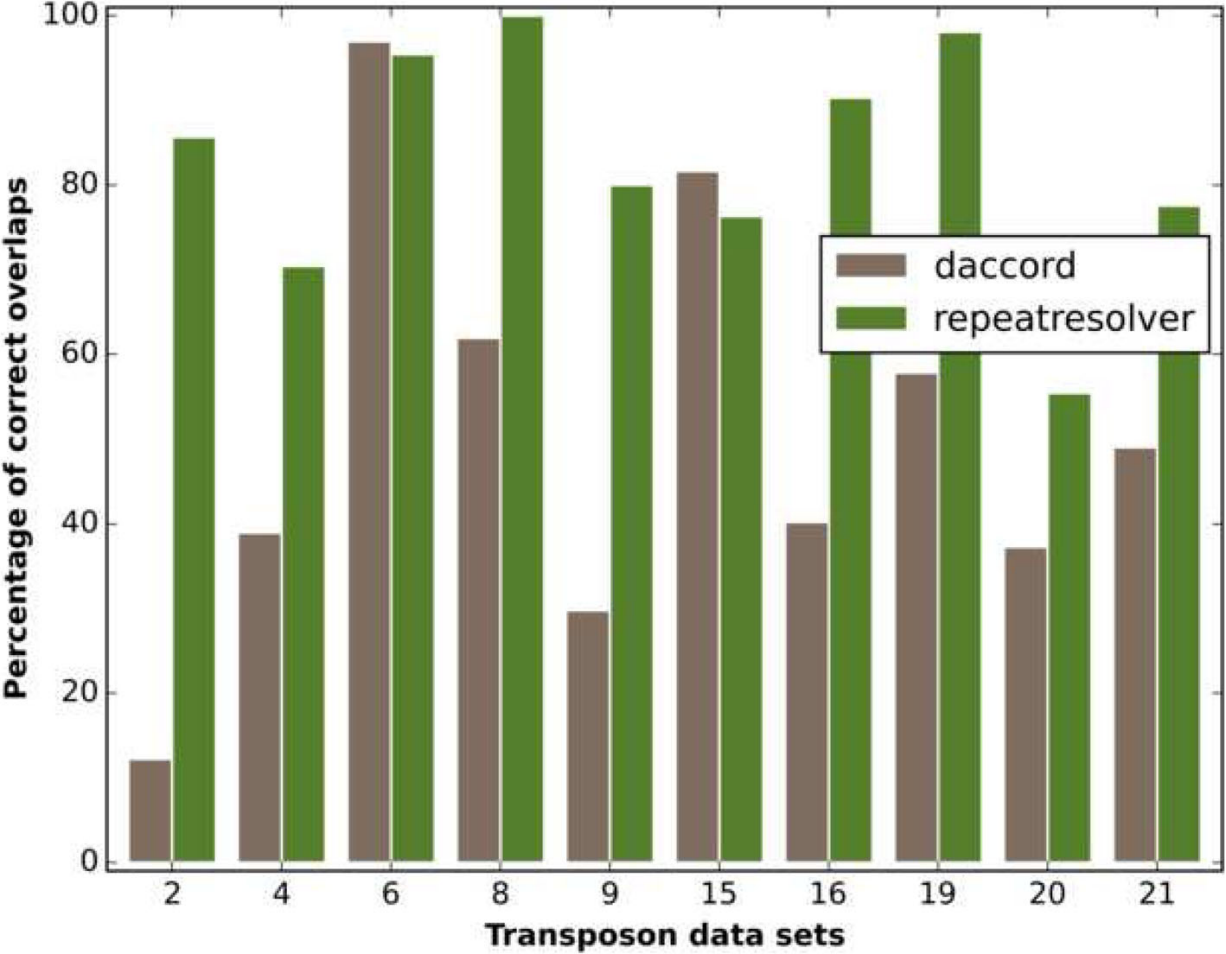
Daccord comparison. We compare the percentage of correct overlaps as provided by Daccord and RepeatResolver for all transposon data sets with fewer than 30 repeat copies

In Fig. 6 we show the percentage of overlapping reads that match the repeat copy of the underlying read for both pipelines and for all transposon data sets with fewer than 30 copies. Daccord shows excellent results, slightly outperforming RepeatResolver, in two out of ten data sets, proving the method sound. In the other 8 data sets, however, RepeatResolver outperforms Daccord. This leads to an average accuracy difference over all 10 data sets of more than 30%, with an average accuracy of 82.9% for RepeatResolver and an average accuracy of 50.6% for Daccord.

## Discussion

Overall our results indicate that, as long as sufficient signal is contained within the data, our novel algorithms are capable of resolving repeats with extremely high copy numbers. This even holds when the resolution has to proceed over many steps to span repeats that are several tens of thousands bases long. While the empirical transposon data shows that not all repeat sequences in genomes are likely to be resolvable, it also indicates that our method is capable of increasing accuracy of genome assemblies.

Our comparison with the Daccord-pipeline shows that our method is superior in accuracy to a current state-of-the-art repeat resolving method for PacBio data, while at the same time remaining computationally feasible for repeat families with a significantly higher number of repeat copies.

## Conclusions

Current long-read genome assemblers cannot resolve extensive repeat regions, because they do not extract distinguishing variants of the different repeat copies. In this paper, we showed that the variant extraction is computationally feasible as a post-processing step. Additionally, we introduced several novel algorithmic ideas to accurately distinguish repeat copies on the basis of the extracted variants.

Extensive empirical assessments show that our work opens up possibilities of substantial improvements in assembly contiguity. Assembly contiguity is especially relevant for highly repetitive plant genomes and for the investigation of structural patterns and variants within genomes. The integration of our repeat resolving tools into the workflow of existing long-read assemblers is the topic of future work.

## Additional file

Additional file 1 Contains additional figures, explanations and details about the algorithms, comparisons and data sets. (DOCX 8737 kb)

## Abbreviations

MSA: Multiple sequence alignment
