# Supplementary material for: Resolving repeat families with long reads
Source: BMC Bioinformatics. 2019 May 9;20:232. doi: 10.1186/s12859-019-2807-4 (PMC6506941; doi:10.1186/s12859-019-2807-4)
Supplement: Supplementary file 1 — Contains additional figures, explanations and details about the algorithms, comparisons and data sets. (DOCX 8737 kb) [file 12859_2019_2807_MOESM1_ESM.docx]

**Resolving repeat families with long reads: Supplementary information**


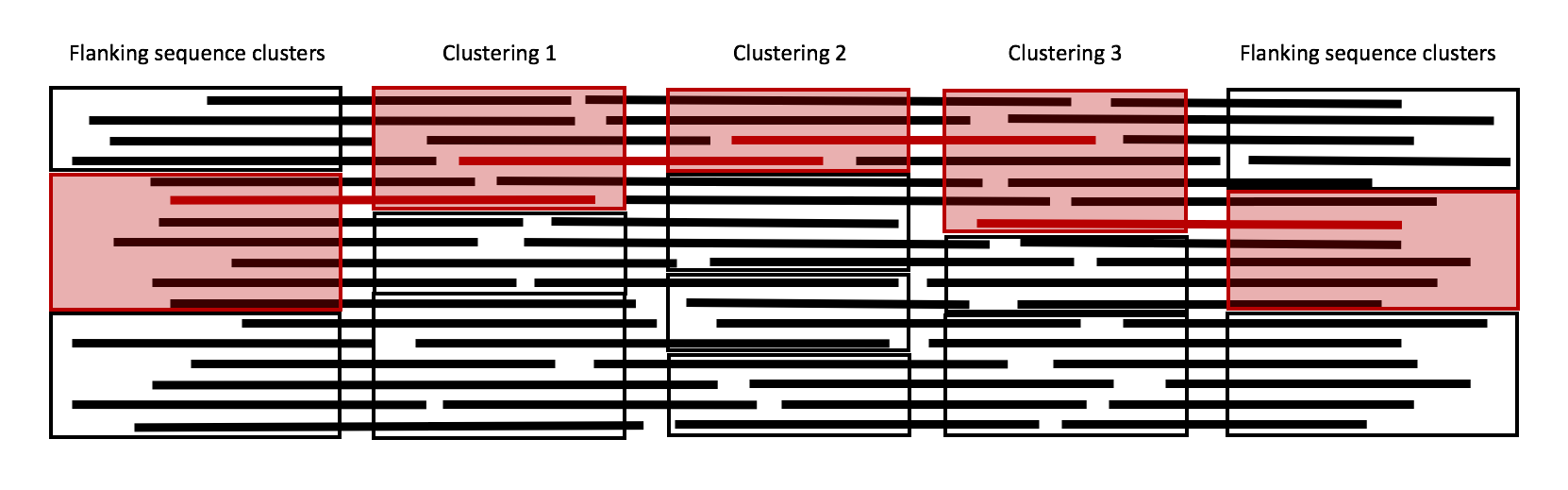


**Figure S1.** A multiple sequence alignment of reads sampled from a repeat family. The unique flanking sequences and sections of the repetitive sequence are clustered with the methods described in this paper. Clusters of flanking sequences are linked by calculating the probability of a randomly sampled path (in red) from cluster to cluster via shared reads to connect the flanking sequence clusters.


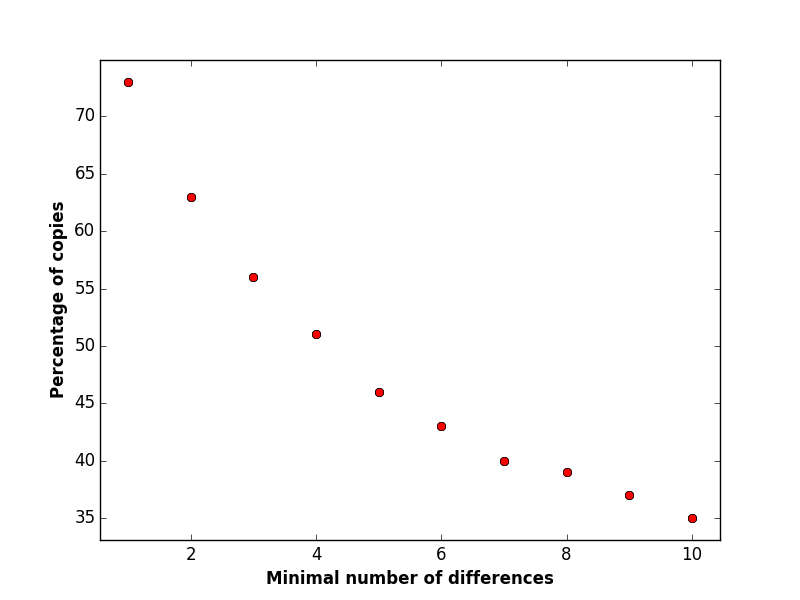


**Figure S2.** The overall percentage of copies in all transposon data sets which exhibit a minimal number of differences compared to the other copies.


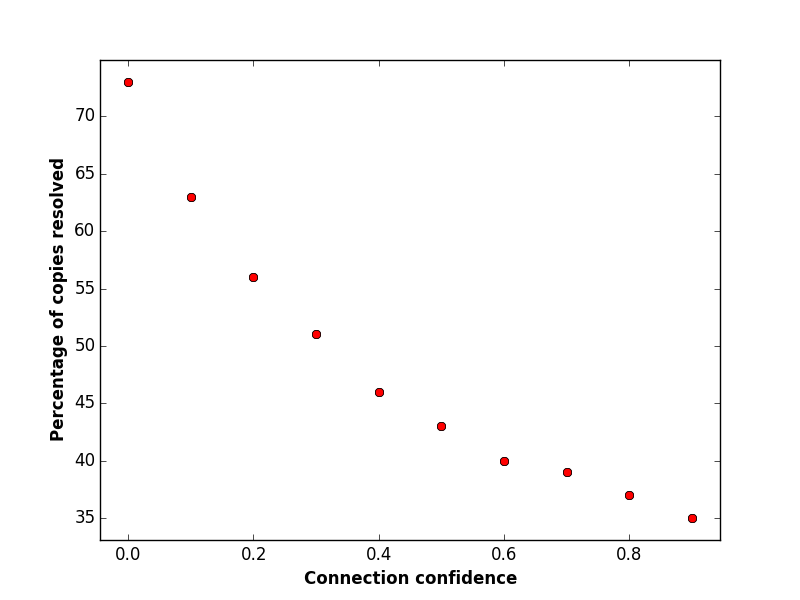


**Figure S3.** The overall percentage of resolved copies in all transposon data sets with a connection confidence above a specific threshold.

**S4: Calculation of the expected differences between pairs of copies in the distributed data set**

In the case of the distributed variant data set we add 3*(x/100)*copy length variations to a randomly chosen subset of the copies with uniform random size. The probability of two sequences not sharing a specific variation can be calculated via $\int_{0}^{1} 2\left( r\times\left( 1-r \right) \right)dr=1/3$ and shows that the expected difference between two copies is x%.

**S5: Applying the daccord/split_dis pipeline to the transposon data sets**

The program split_dis infers coverage information under certain read length and sampling distribution assumption. Our transposon test data sets do not satisfy these assumptions, as the unique flanking sequences have been removed and only repeat sequences of equal length remain. In consultation with the author of Daccord, we change the parameter **uint64_t const dprime = (avgreadlen - phasethres) / a;** to **uint64_t const dprime = d;** in the file **split_dis.cpp**. This circumvents the inference of coverage information by directly providing the correct value.

We run split_dis with the –d20 parameter, again after consultation with the author of Daccord.

Apart from these minor tweaks we follow the documentation in the usage of daligner, daccord, computeextrinsicqv, and split_dis.

Table S6: Properties of the transposon data sets A)

| **No.** | **Symbol** | **Template**  **length** | **Average length** | **Coverage** | **Transposon type** |
| --- | --- | --- | --- | --- | --- |
| **0** | Idefix | 7411 bp | 7215 bp | 37 | Retroviral elements |
| **1** | 17.6 | 7439 bp | 7380 bp | 45 | Retroviral elements |
| **2** | 1731 | 4648 bp | 4672 bp | 45 | Retroviral elements |
| **3** | 297 | 6995 bp | 6850 bp | 51 | Retroviral elements |
| **4** | 3S18 | 6126 bp | 6093 bp | 37 | Retroviral elements |
| **5** | 412 | 7567 bp | 7538 bp | 46 | Retroviral elements |
| **6** | aurora-el. | 4263 bp | 4479 bp | 51 | Retroviral elements |
| **8** | BS | 5142 bp | 5168 bp | 54 | Non-LTR retrotransposons |
| **9** | Burdock | 6411 bp | 6371 bp | 49 | Retroviral elements |
| **10** | copia | 5143 bp | 5201 bp | 48 | Retroviral elements |
| **11** | Doc | 4725 bp | 4762 bp | 48 | Non-LTR retrotransposons |
| **12** | F-element | 4708 bp | 4690 bp | 46 | Non-LTR retrotransposons |
| **15** | G-element | 4346 bp | 4361 bp | 48 | Non-LTR retrotransposons |
| **16** | gypsy | 7469 bp | 7481 bp | 35 | Retroviral elements |
| **19** | HeT-A | 6083 bp | 6128 bp | 51 | Non-LTR retrotransposons |
| **20** | I-element | 5371 bp | 5376 bp | 41 | Non-LTR retrotransposons |
| **21** | jockey | 5020 bp | 5048 bp | 43 | Non-LTR retrotransposons |

Table S6: Properties of the transposon data sets B)

| **No.** | **Copy number** | **Ground truth coverage** | **Daccord runtime per read** | **Number of reads compared** | **Average comparison clustersize** | **Number of reads** |
| --- | --- | --- | --- | --- | --- | --- |
| **0** | 37 | 23 | None | None | None | 1388 |
| **1** | 49 | 27 | None | None | None | 2240 |
| **2** | 25 | 27 | 1d19h | 18 | 15.5 | 1297 |
| **3** | 44 | 35 | None | None | None | 2207 |
| **4** | 16 | 29 | 16h | 29 | 20.4 | 605 |
| **5** | 34 | 26 | None | None | None | 1575 |
| **6** | 7 | 41 | 3m | 289 | 16.1 | 359 |
| **8** | 9 | 41 | 1h12m | 73 | 19.0 | 487 |
| **9** | 13 | 33 | 13h | 18 | 14.2 | 646 |
| **10** | 89 | 30 | None | None | None | 4284 |
| **11** | 135 | 33 | None | None | None | 6480 |
| **12** | 157 | 33 | None | None | None | 7317 |
| **15** | 8 | 29 | 8m | 239 | 20.5 | 391 |
| **16** | 12 | 18 | 3h49m | 66 | 16.3 | 423 |
| **19** | 5 | 32 | 35m | 164 | 20.4 | 257 |
| **20** | 20 | 30 | 14h | 17 | 30.3 | 832 |
| **21** | 22 | 32 | 1d17h | 12 | 62.8 | 961 |

Table S7: Properties of the simulated datasets

| **No.** | **Type** | **Copy differences** | **Coverage** | **Repeat length** | **Read error** | **Resolved copies (multi)** | **Resolved copies (single)** | **Connection confidence (single/average)** |
| --- | --- | --- | --- | --- | --- | --- | --- | --- |
| **0** | Equidistant | 0.1% | 30-40X | 30kb | 15% | 1.0% | 100.0% | 14.4 |
| **1** | Equidistant | 0.5% | 30-40X | 30kb | 15% | 99.0% | 99.66% | 97.6 |
| **2** | Equidistant | 1.0% | 30-40X | 30kb | 15% | 96.0% | 99.33% | 98.1 |
| **3** | Distributed | 0.1% | 30-40X | 30kb | 15% | 4.0% | 97.66% | 37.1 |
| **4** | Distributed | 0.5% | 30-40X | 30kb | 15% | 96.0% | 98.66% | 71.3 |
| **5** | Distributed | 1.0% | 30-40X | 30kb | 15% | 100.0% | 100.0% | 99.4 |
| **6** | Tree-like | 0.1% | 30-40X | 30kb | 15% | 44.0% | 83.83% | 52.6 |
| **8** | Tree-like | 0.5% | 30-40X | 30kb | 15% | 99.0% | 99.83% | 98.5 |
| **9** | Tree-like | 1.0% | 30-40X | 30kb | 15% | 95.0% | 99.33% | 98.3 |

**S8: Benchmarking MARVEL and Canu repeat resolution on simulated data**

Our simulated datasets consist only of reads sampled from a repeat copy with 10kbp flanking sequence on both ends. To benchmark Canu, we double the flanking sequences to avoid possible confusion stemming from reads spanning unique sequence between reads, and concatenate all repeat copy sequences into a 7mbp sequence. We then sample PacBio-typical reads from this sequence with an error rate of 15% and a coverage of 40X.

Canu and MARVEL are run on the resulting datasets for the repeat types *tree-like*, *distributed* and *equidistant* with a copy difference of 1%.

Canu is run with the command:

./canu -p Distributed -d /Canu genomeSize=7.0m -pacbio-raw /Canu/Distributed_1perc_30000kb.fasta

For the tree-like dataset the flag stopOnLowCoverage=0 was necessary, likely because the gradient of copy differences in that dataset made read correction difficult. The distributed dataset results in 97 contigs, the equidistant dataset results in 106 contigs, and the tree-like dataset results in 122 contigs.

MARVEL is run with the dedicated do.py script. The distributed dataset results in 100 contigs, the equidistant dataset results in 101 contigs, and the tree-like dataset results in 101 contigs.

| 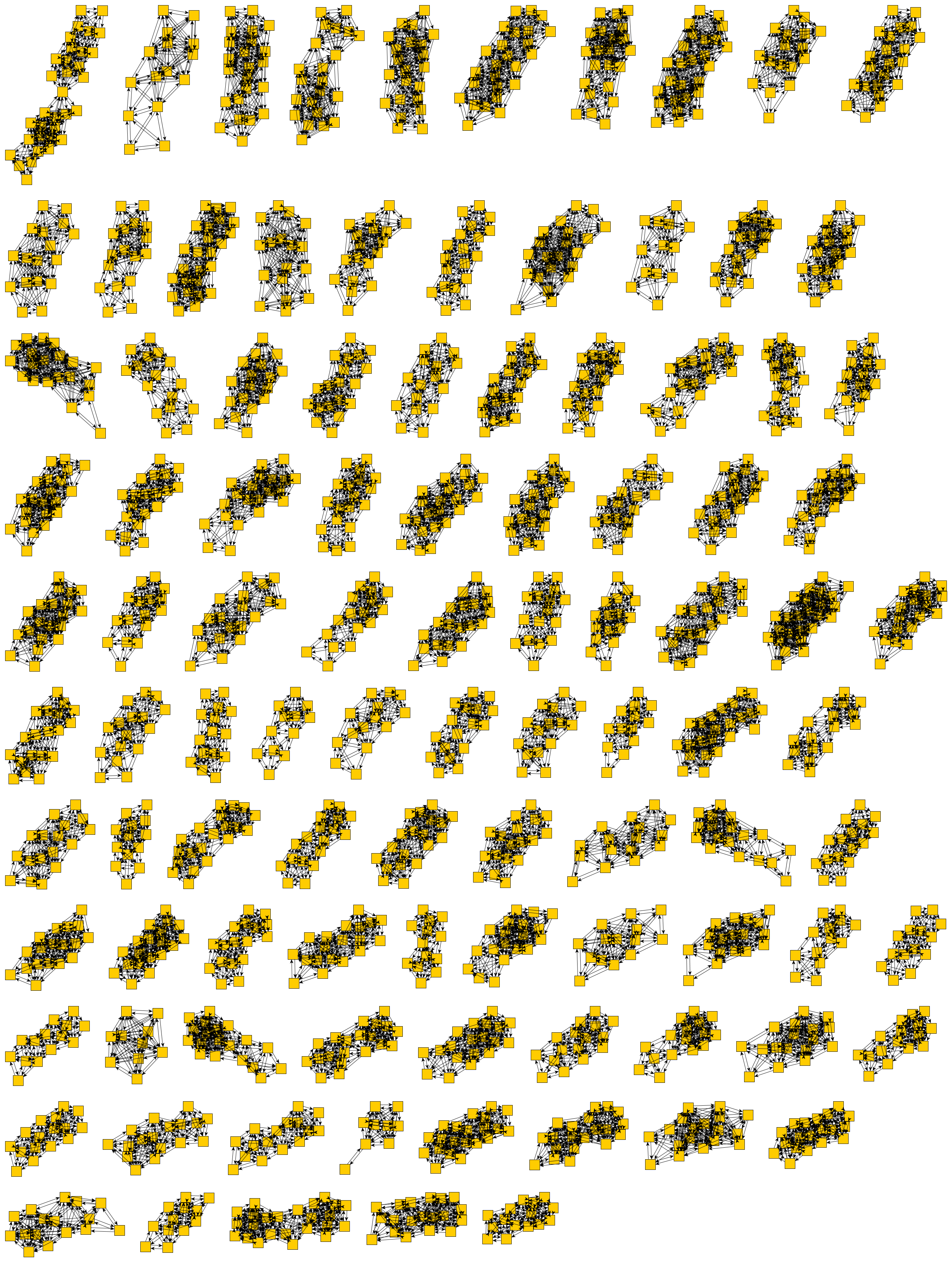 | 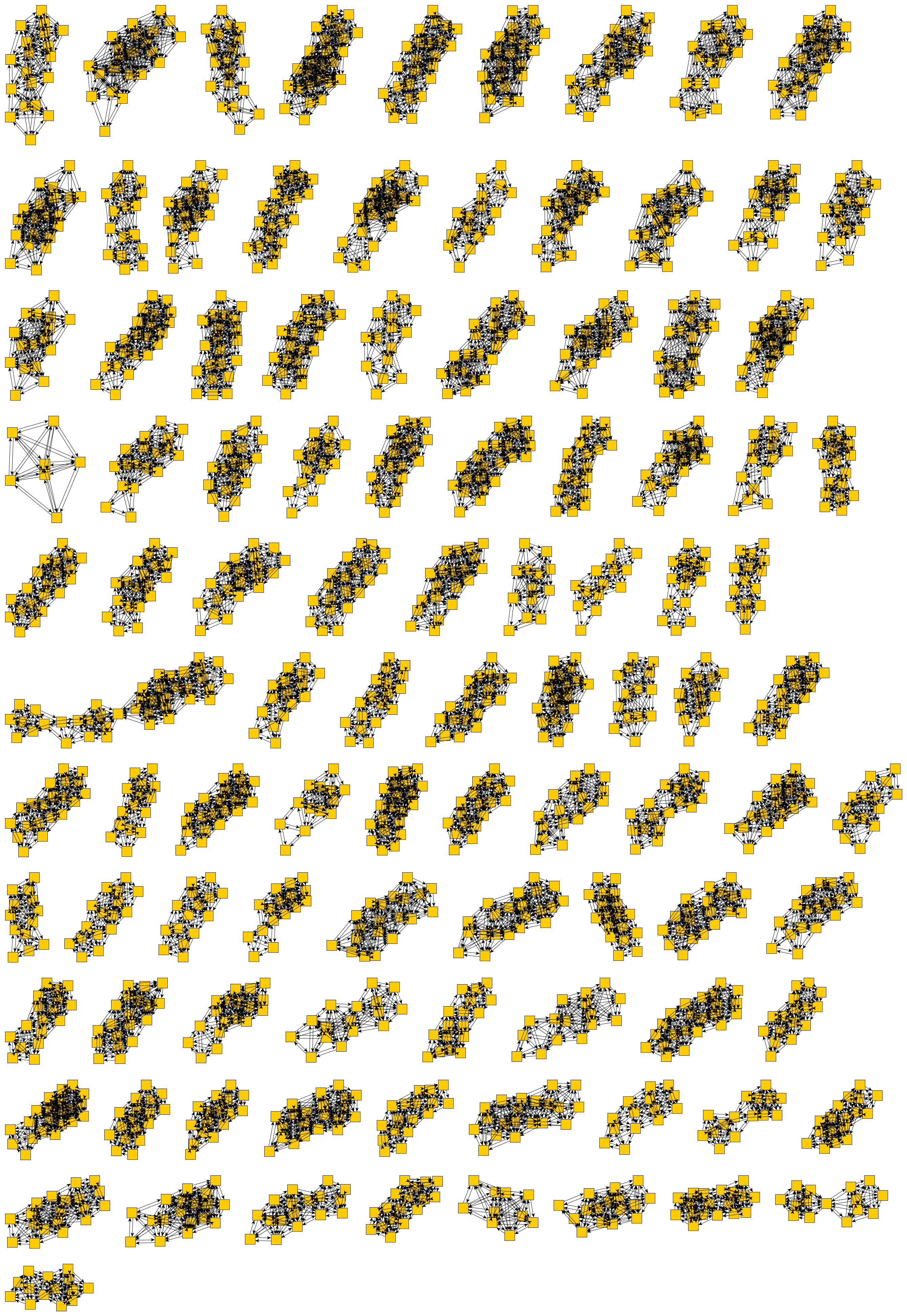 | 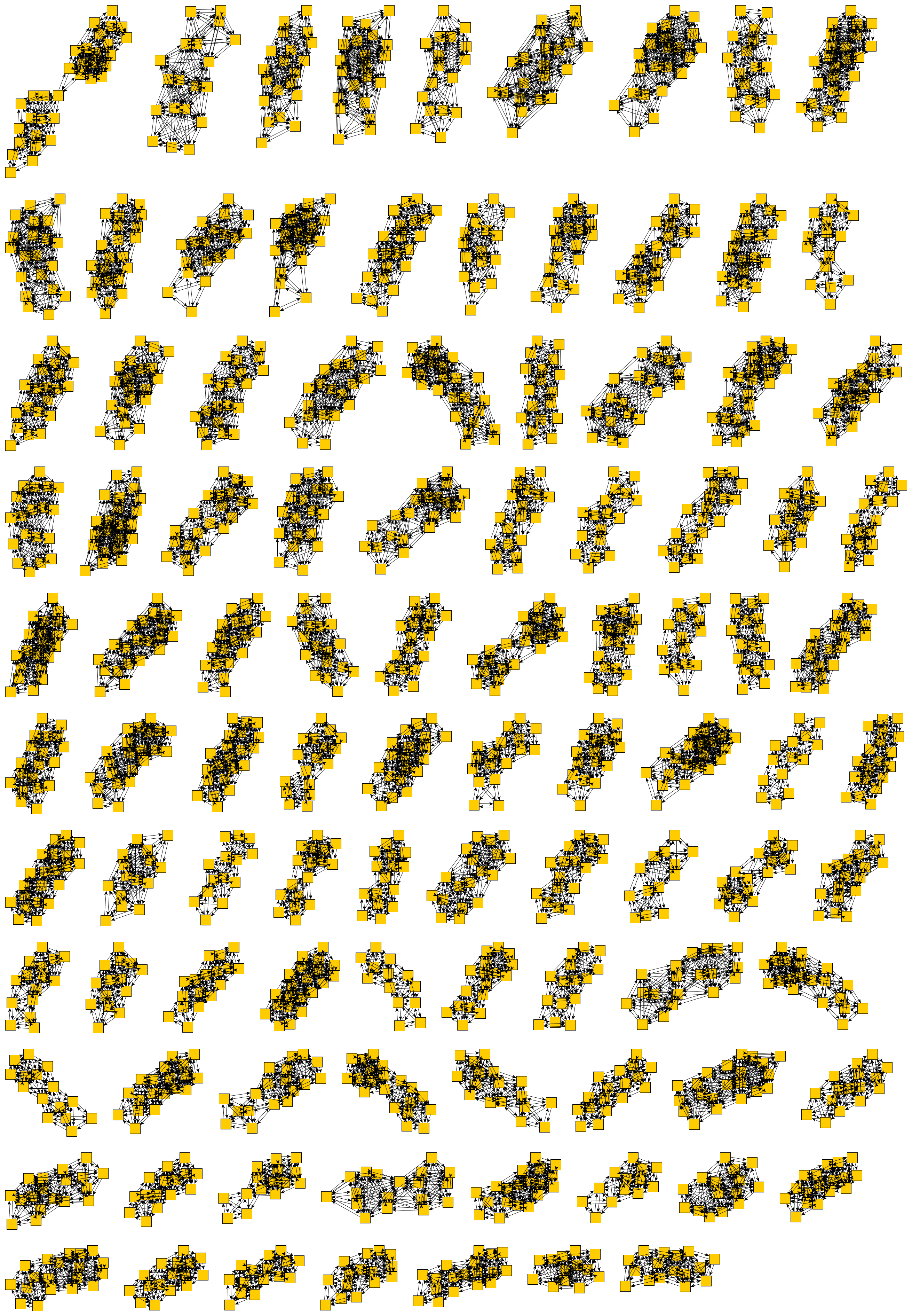 |
| --- | --- | --- |

**Figure S9.** The MARVEL assembly graphs of our simulated data sets (distributed, equidistant, tree-like) show that MARVEL cannot resolve 30kbp repeats with 1% copy differences.
